# Nature in Disease

**Published:** 1853-02

**Authors:** 


### ECLECTIC DEPARTMENT,

### AND SPIRIT OF THE MEDICAL PERIODICAL PRESS.

---

*Nature in Disease.* An Address delivered before the Norfolk, (Mass.) District Medical Society, at the Annual Meeting, May 12th, 1852, and printed in accordance with a vote of the Society.* By B. E. Cotting, M. D., of Roxbury.

We give insertion in our pages to the following address, because it seems to us well calculated to direct the thoughts of the reader in a direction too

* In yielding, reluctantly, to the vote of the Society and the solicitations of friends, it is hardly necessary to remark that no one estimates more highly the value of a thoroughly rational, scientific treatment of disease, than the author of this essay. Such treatment is not only highly beneficial, but all-important. It is the routine, unscientific, reasonless and unnecessary medication, overlooking the real nature and tendencies of disease, which he deprecates.

often lost sight of by the medical practitioner. In connection with the subject matter, and tenor of the address, the note appended by the author is to be considered. While it is important in the pursuit and practice of medicine to study and appreciate the natural tendencies of disease toward recovery, it is equally important to study and appreciate the operation of remedies in arresting diseases, abridging their duration, divesting them of their intensity, and disposing to a favorable termination. In proportion as we advance in our knowledge of the natural history of diseases, it is true that the instances become more and more numerous in which the active interference of the physician is shown to be unnecessary, and, if so, of course injurious; but the duty of interference, under certain circumstances, is not on that account rendered less imperative. Discrimination and judgment in withholding as well as wielding potent remedial agencies, are invested with more and more importance in proportion as our knowledge advances. True boldness in medical practice is frequently evinced in the resolution and persistency with which a "masterly inactivity" is displayed; and, on the other hand, it is conspicuous in the prompt efficient application of powerful medication. The "expectant method" is not blindly and uniformly adopted, more than heroic remedies are indiscriminately resorted to by the judicious practitioner. We make these few remarks that we may not, more than the author, be misconstrued by the publication in our journal of the following address. Regarded in a proper light, with due qualifications in their general application to medical practice, we conceive that the views submitted by Dr. Cotting may be read with profit, as well as interest.—Editor Buffalo Med. Journal.

Notwithstanding the rapid progress of medical science in these latter days, and the great advances the present has made over past ages in freeing our profession from the mysticisms which have ever enveloped it, it is still to be feared that too many of our fraternity set out upon their professional career indelibly impressed with Mr. Bagges's notion, that "disease is a certain noxious something, to be destroyed by medicine as an acid by an alkali;" and when, like Dr. Labell, they have treated their patients to "leeches, blisters, antimony, opium, ether, ipecac., colchicum — lotions, fomentations, and liniments"—they, like him, take good care to impress upon the convalescent that these medicines have cured the disease by putting a stop to it! Believing this themselves, they indoctrinate their patrons, and through them the public, with the same idea. But it must have early struck the attentive student, as it may now-a-days even the superficial observer, that under various and conflicting methods of treatment many diseases come to about the same general results—about the same relative number of recoveries and failures. For a longer or shorter period, the most diverse theories, as of Cullen, and Brown, of Broussais, and Rasori, and others of a lesser note, have claimed and held pre-eminence. During its reign, each has not only been considered superlatively successful, but boasted its unrivaled cures. Under each, patients recovered in sufficient numbers to enable its followers to predict its universal adoption. That many also died, though drugged in strict accordance with the prevailing and supposed infallible theories, as well as under other methods of treatment, is sufficiently evident from the fact that these systems lost the confidence they once obtained, and now only remain in the memories of our older practitioners, or serve to amuse those whose curiosity leads them to search the records of past hypotheses. No system has now such unquestioned sway, as those of Cullen and Brown with our fathers. We are now in an unsettled state—in transition from hypothetical to more rational methods. The doctrine of "nature curing diseases," so full of baneful influences on the practice of physic in the opinion of Cullen and his followers, has been stripped of most of its supposed dangers, by the present generation, and is again in the ascendancy. The present period is remarkably favorable for more extended and more correct observations in this regard, and it is to be hoped that it will not pass unimproved by the profession.

The science of therapeutics, though freed of many of its absurdities, has not yet made great positive advances when compared with other branches of medical knowledge. Nevertheless, the recent results of a more exact pathological anatomy, registered and counted, have not been without their salutary effect upon the treatment of diseases. Sixteen years since, Dr. Bowditch's translation of that incomparable work of Louis on Typhoid Fever, was distributed to the members of the Massachusetts Medical Society. Many a doubting glance was cast over its pages, and grave and respected elders were then heard to remark to each other and to the bystanders, "that it would be a disgrace to any New England physician to treat fever as recorded in that work." The vigorous—to call it by no harsher name—the vigorous treatment then and previously pursued in this neighborhood for typhoid fever, had *done so much* that the expectant method, therein alluded to, seemed doing nothing indeed. Venesection, emetics, cathartics, blisters and mercury, the remnants of English heorics, stood in strange contrast with the milder trifles, the barley-mixture and gum-syrup of the French hospitals.

The previous year, Dr. Bigelow delivered his admirable discourse on self-limited diseases, before the same society. The doctrines of that discourse fell like an exploding bomb-shell into the camp of those who had taught their patients, and probably themselves believed, that they had *broken up* unnumbered cases of fever by a master-stroke in the commencement, or had cut short their triumphal progress by some wonderful exploit of professional strategy. Many went away sorrowful at the doctrine—some at such heresies in high places, and some fearful perhaps that if disease had not suffered at their hands, the patient certainly had. The right spirit, however, was awakened. Accurate investigations were made and recorded. Autopsies, rigorous and general, were instituted anew; and the result has been that an entirely new view of the history and pathology of typhoid fever has since prevailed. And, whether the redness and ulceration of Peyer's patches stand in the relation of cause and effect, or neither—a constant coincidence of these phenomena with this fever, and the increasing belief of its self-limited nature, have been sufficient to remodel the plans for treatment. This has been done so effectually, that it may now be doubted whether it would not be a disgrace to any one of us not to recognize the principles established by Louis, in our treatment of this and similar diseases.

Valuable as these advances have been, the practical inquirer has other and equally important questions to ask of the observer. Disease has been noted, registered, and counted, under various forms of treatment—what would its history and course be, if left to itself, under no treatment at all, without the administration of any drugs, with a view to cut short or even to mitigate its progress? For this question must receive a distinct and definite answer, from the observation of a sufficient number of cases, before the real value of any method of treatment can be truly estimated.

It may be said, and with truth, that this is a difficult question to decide—that single cases vary greatly in character—that the constitution and state of the patient are not the same, for any two individuals—that in its tendency, severity and complications, each case differs from every other. But all this does not alter the proposition. From such cases we are constantly proclaiming the value of certain remedies, and deducing plausible theories of treatment. Aye, but the experiment—who will be bold enough to try it? The sin of omission in practice is the unpardonable of offences. To have *tried everything that could be thought of* is the impregnable retreat of the baffled practitioner, and a balmy sedative to the bereaved. Nevertheless, until the benefits of the prescription over its omission be known, the administration of a drug is as great and as hazardous an experiment as the withholding of it. Who can say with truth that it is not even more dangerous? The popular reasoning, that "it will do no harm if it does no good," may be sufficiently satisfactory to ignorant and officious bystanders, who seem sometimes to literally revel in an opportunity to crowd a patient's stomach with multifarious mixtures, and to load his person with offensive masses; but it will hardly bear the test of ordinary common sense. The suffering individual may prefer the trial at any risk, under the irksomeness of debility or the pangs of disease; but a compliance with his wishes, followed by recovery, is not proof positive that he has been benefitted thereby.

A violent fever sets in—you bleed the patient, and administer powerful drastics. In a few days he is well. Has the disease been *broken up?* Might he not have recovered equally well and speedily had he never seen you, or your supposed remedies? Cases of recovery under similar circumstances, without interference, are not infrequent. And until the question can be decided on a large scale—until the degree of probability in a given case can be shown from multitudes of observations, the value of your interference, for good or for evil, must remain uncertain and problematical.

Now, hundreds of cases of typhus fever have been submitted to the most thorough expectant or let-alone treatment; and it has been found that so far as duration of the disease is concerned, the results were quite favorable. Cases commencing with most violent symptoms of inflammation, delirium, &c., &c., have subsided, after a day or two, and convalescence been fully established in less than a week. It has been found that the natural duration of the disease is from three to nearly or quite one hundred days—some of the longer cases having commenced or terminated so gradually as to render precision to a day impossible, and the shorter ones resembling, as far as they ment, those which proved of longer continuance and dangerous severity. By far the largest number were convalescent in less than twenty days. In severity of daily and progressive symptoms, these cases compare favorably with equal numbers of others under the various and ordinary treatment of competent practitioners. In general results, these cases presented a decided amount of recoveries over those in which an active, or heroic, treatment was employed.

We may not be able or willing to adopt such a course for an individual in private practice; for, as has well been remarked, "such treatment may do for armies, where one man isas good as another; but does not answer for individuals, by nature prone to over-estimate their personal consideration." Still, until the principles be established, by which the individual may securely have just that degree of treatment suited to his distress and danger, better than they now are, the results of such investigations must have a beneficial influence. Let every opportunity of observing a case of fever, undisturbed by drugs, be improved by each one of us. It cannot fail to add to our knowledge of the real nature of our disease, and, perhaps, may save some of our patients from unnecessary suffering; for, although some of us may be wandering amongst infinitesimals, the most of us in medicine, even now, like the rich in their wealth in Hesiod's time, "do not know how much better a half is than a whole."

The truly expectant plan has also been tried in the treatment of scarlet fever—in fewer cases, but with very similar results. This disease is admitted on all hands to be self-limited, and no one pretends to break it up. Yet there are indications from all quarters, especially from such observations as those alluded to, that even in this day of small doses, professional overdosing is a great obstacle to the speedy and perfect recovery from this complaint. These cases of too much interfered happen the more frequently, where the great anxiety of influential friends, stimulating the too ready attendant, exacts a multitude of appliances and a legion of remedies—that there may be abundant evidence of "doing something" for the victimized patient. How much the probabilities of recovery have thus been diminished; how many, if not fatal, at least severer sequelæ have thus been entailed upon the sufferer; how many broken constitutions, what impaired vitality, and greater susceptibility to noxious influences; how many weaknesses in protean forms have thus originated; how many a fatal termination has thus been directly induced, we may never know. We may, however, taking heed to such suggestions, be less anxious to invent new prescriptions and appliances, than to dispense with many now usual and popular, lest perchance it some time turn out to our mortification that the disease, in our day and generation, is really less formidable, as nature forms and develops it, than as modified and complicated by the ordinary interferences of art.

The natural history, progress and tendency of dysentery, if carefully reinvestigated, would form no mean addition to our professional aquisitions. That this disease tends to recovery, and is actually recovered from, in sufficient number of cases to inspire confidence in the treatment, under all varieties of practice, from the most heroic drastics to the most imaginary doses—the treatment by opium and astringents not having warmer supporters than that by repeated potions of castor oil; nor these than that by billionths of a grain of corrosive sublimate—and that we so often hear practitioners complaining that it is so very "obstinate" or unyielding to remedies, this or that season, are sufficient indications that it is self-limited, and defends itself, as best it may, against excessive medical interference. That, as in typhus, scarlatina, and other exanthems, a person having experienced one attack of this disease is thereby protected against a second, though not so certainly proved, is not improbable from recent observations. The subject throughout possesses unusual interest, and is deserving of attentive revision.

In 1835–6, Dr. James Jackson caused to be translated and published, Louis's work on "Bloodletting in some Inflammatory Diseases, and on the Influence of Tartarized Antimony and Vesication in Pneumonitis," and added thereunto his valuable collections of cases from the records of the Massachusetts General Hospital. He was induced to publish this work, he says, by the deep impression which Louis's results, so little in accordance with the general opinion, had made on his own mind. And he candidly admits, after re-examining the cases referred to, that "it would seem to be of less importance whether our patients were bled or not, than whether they entered the hospital early or late." That is, comfortable apartments and attentive nursing exercise a greater influence over this disease, than all the boasted powers of bloodletting then so universally relied on. Well might he add, that such results "will, no doubt, surprise many, if not most medical men." They did surprise the profession; and the treatment of pneumonia now is quite a different thing from the treatment of the same disease fifteen years ago. Whether venesection is now sufficiently employed in pneumonia, or not, is a question I cannot answer; but certain it is that the average of fatal cases treated without it, in this vicinity, does not exceed, but rather falls short of, that stated by Dr. Jackson for the cases so treated in the Mass. General Hospital. At the time of the publication of the work alluded to, it was the practice, in this section of the country at least, to administer antimony in pneumonia to constant nausea—to tolerance, so called. This was a very happy expedient for the routine practitioner—so simple a thing was it to mingle the drug in the customary proportion of water, and so satisfactory a matter was it to nurses and friends to find sweet solace in the frequent administration of the mixture. But the poor patient—who that has once seen can ever forget the involuntary shudder, nay, the inexpressible horror, when the repulsive draught was again and again offered? Nor was the evil always confined to the administration of the supposed remedy. "Redness, soreness, and even pustules were produced in the fauces," admits Dr. Jackson. Yes, and autopsies revealed pustules throughout the intestinal canal, even where tolerance had not been exceeded. I well remember the subdued undertone in which such facts were whispered about among the profession; and the trembling hesitancy with which antimony was subsequently administered by those whose faith in it could not be shaken, though they were ready to admit an unaccountable irritability of the mucous membrane in some idiosyncrasies. How much the patients unnecessarily suffered by this and other equally harsh medicines for this disease, will probably never be accurately estimated—how many were relieved of their distress, or restored, in consequence of such practice, will remain equally a subject of conjecture. One thing is certain, that many distinguished practitioners thought and taught that they effected "remarkable cures" by such a course of treatment. And another thing is now not less certain, from the testimony of most respectable members of the profession, who have watched, expecting to prove the contrary—that pneumonia, even in the severer forms, may pass, with perhaps equal certainty, through all its stages to perfect recovery, under the administration of infinitesimal atomies.

Perhaps no disease, in this vicinity, is more dreaded by parents, and practitioners also, than membranous croup. Certainly none requires more assiduous attention, and offers less prospect of ultimate success. We now speak of the membranous disease, and not of those so-called spasmodic or catarrhal affections generally classed with it. These latter, though often violent and alarming in the outset, are comparatively harmless, and ought no longer to be called by the terrific name of croup, with which they have little or no affinity.

Sixteen years since it was taught, from the lips of undoubted authority, that "croup is death." Its great fatality, its great frequency in certain localities, and the insidious nature of its attack, have made it the subject of observation by many anxious inquirers, who, of late, have added much to our knowledge of its nature and history. It has been found that exudatory inflammations (affecting chiefly, but not exclusively, the larynx, trachea and bronchiæ) spread invariably from above downward, and not in an opposite direction; that if it commence in the trachea it may descend into the bronchiæ, but will not mount to the larynx; that with nursing children false membranes are not infrequent in the fauces only, and that the liability to descend into the larynx increases in proportion to the age of the child; that in adults, on the contrary, false membranes are, except in rare cases, chiefly confined to the smaller bronchiæ. It has been found, also, that the membrane itself is of a peculiar nature—a tissue of elastic fibres, longitudinally arranged; the fibres smooth, and in no degree transversely striated. Great elasticity is one of its chief characteristics. It is inorganic in its nature, or so much so that it never tends to organic union with the subjacent tissues. In proportion and as soon as the inflammation begins to abate, it separates, and, by irritating, causes itself to be thrown off. It may be re-formed a second, or even a third time. Though generally considered the result of a peculiar species of inflammation, it certainly obtains in other parts of the system, and moreover (from which we may learn a lesson of caution in our treatment) fatal exudations, similar in many if not in all respects, have been known to take place in previously healthy larynges from the accidental inhalation of caustic vapors.

It is believed, from careful investigation, that death is not oftener due to the obstruction of the membrane than to the weakened or paralyzed action of the muscles which open the glottis—though spasm seems to be most dreaded by attendants generally. And further, observation has shown that cases of undoubted recovery, with expulsion of the membrane, have taken place under treatment by calomel to excessive salivation, emetics to cruel barbarity, caustics to distressing peril, more frequently under the milder process of anodynes and watery vapor, sometimes under imaginary doses; and, lastly, without any medical treatment, real or pretended—so that it must be set down among the self-limited diseases, with a natural tendency, though feeble it may be, toward recovery.

These few diseases have been adduced, among others that might be cited, to illustrate the position assumed, and to indicate the kind of observations we would urge. Such observations any one of us may make. They are easier, and will be more serviceable to ourselves and the profession, than attempts to solve the mysteries of disease by pathological dissections. These, though more generally insisted on, and certainly never to be neglected, often require most skilful hands and the most patient examinations of the practiced, and the numbers of cases which only large cities can supply; but the other is forced upon us at the bedside of every patient. No one can over-estimate the importance of correct knowledge on this subject. Without it we shall ever be uncertain as to the *real* value of any therapeutic interference. The fear of not doing enough may deter us; but we have seen how much the best physicians have formerly erred in their implicit reliance on powerful medicines to shorten disease and to restore health. And we know that the natural tendency to recovery under simple nursing, or under imaginary doses, is at least as great as under the formidable heroics of former times. "When I came upon the stage," wrote a few days since a venerated friend, who last year entered on his second half-century of active practice—"when I came upon the stage, whatever might be the differences of opinion about the nature or origin of the disease, there was none at all about the treatment: the first day an emetic, the second a cathartic—just as regular as the first and second bells for meeting on Sundays. Over and over again, during my pupilage, I have heard the patient say to my teacher, "O doctor, I know I ought to have sent to you before, but I did *so dread* to take an emetic!" And this dread of seeing the doctor for fear of an emetic, was founded on woful experience—the one was as sure as the other. And such doses—Lord save us! Nothing short of the indomitable spirit and power of that strong race could have carried the Pilgrim Fathers through their trials, or their descendants through their struggles with such Herculean medical practice."

Thus saith my friend—and at the present day may it not be that we are standing in a similar position toward those who may come fifty years after us; and this the more likely, as it is an occasional remark of continental visitors, abundantly qualified to make correct observations, and after sufficient experience and intercourse in the country, "that our people are martyrs to drugs and medicines—and this, too, at the hands of the profession."

If we ourselves are not able or willing to make the trial where we feel that experience has given a power to alleviate or to arrest, many of us, if so disposed, may turn to account the cases of our neighbors who honestly deal in infinitesimals. It were better for ourselves, and the science to which we are devoted, to avail ourselves of such opportunities than to waste our time and temper in empty cavilings against their vaunted, but, as we believe, baseless theory. If we need not the instruction ourselves, it is time the public were instructed by us in more correct notions of the nature of disease. So long as physicians teach their patients, directly or indirectly, or allow themselves to suppose, that diseases cannot be removed unless broken up by some masterly exploit, or amazing mystery of art, so long will the profession stand in a false position—so long will it be subject, as in times past, to violent alternations from formidable heroics to mystified trifling—so long will practitioners be doomed to have some of their sickest patients taken from them and placed at the critical moment in the hands of reckless adventurers; perchance to recover under treatment wholly inappropriate or totally inefficacious—so long, also, will medicine be ranked among the uncertain sciences, and its results be classed by intelligent laymen as the offspring of blind chance. With more frequent reference to the natural history of disease, physicians will adopt a less assuming and presumptuous bearing, which, while it serves to make the vulgar stare, brings grief into the hearts of the discriminating. The most celebrated of our profession, ever remarkable for their little reliance on the specific powers of medicine, and always noted for administering the smallest quantities and the mildest forms, have ever been distinguished for modest demeanor and a willingness to admit that they have been merely careful attendants and watchful assistants, nature guiding at the bedside of the sick. Thus we hear an illustrious example of medical lore, after skillfully carrying a patient through a protracted and almost hopeless disease, modestly remarking that he had "visited the lady and the Lord had cured her." And we are not the less impressed to admiration with the renowned skill of that glorious veteran of military surgery, after a successful attendance on a chieftain horribly mangled in battle,

> "Who wrote from Suza's blood-stained field,
> 'I dressed the wound that God has healed.'"

Here, too, in our own day and circle—those of us who were privileged to listen to the teachings of

> "The truest, noblest, wisest, kindest, best,"

of physicians and men, will bear witness to the earnestness with which he deprecated the use of the word *cure* as a result of medical treatment, and the decision with which he excluded it from the Hospital records, adding that in its legitimate sense (to cure meaning to take care of) all such patients had been *cured*, though only a part had *recovered*.

If we read aright the signs of the times, this spirit prevails to a greater extent than ever before in the history of the profession, and is on the increase. It is of good omen—let us bid it God-speed. We need not fear the loss of position and influence by instructing the community in the true nature of our science. The want of such information, and the belief that each disease or symptom has its appropriate and infallible remedy, if the practitioner could only hit upon it, has been the source of infinite mischief—the foundation of professional huckstering, and of vulgar empiricism. The only remedy for such evils, widely felt and sufficiently deplored, is to be found in an earnest and persevering application to investigations such as we have advocated. Such investigations will raise the medical attendant far above the mere prescriber of drugs or the dealer-out of nostrums. They will open his mind to a nobler view of his calling, and give a loftier purpose to his mission. To responsibilities, greater than fall to the lot of other mortals, they will add the necessity of augmenting professonal acquisitions by an enlarged knowledge of collateral sciences. To watch carefully, to study thoroughly, to guide cautiously, will become only the more imperative. Individual labors may thus be increased; but as such investigations are successfully pursued, and the knowledge of the real nature of diseases better known and promulgated, the relations between physician and patient will rest on a more rational basis, the profession will reach a higher elevation and take a firmer hold on the confidence of the people, than it has ever yet attained; and its members will be saved from the reproach now sometimes cast upon them, that they have been "ever learning, but never able to come to the knowledge of the truth."

